# Human Mesenchymal Stem Cells on Size-Sorted Gelatin Hydrogel Microparticles Show Enhanced In Vitro Wound Healing Activities

**DOI:** 10.3390/gels10020097

**Published:** 2024-01-26

**Authors:** Derya Ozhava, Cemile Bektas, Kathleen Lee, Anisha Jackson, Yong Mao

**Affiliations:** 1 Laboratory for Biomaterials Research, Department of Chemistry and Chemical Biology, Rutgers University, 145 Bevier Rd., Piscataway, NJ 08854, USA; do311@chem.rutgers.edu (D.O.); cemile.bektas@rutgers.edu (C.B.); kwl49@scarletmail.rutgers.edu (K.L.); atj43@scarletmail.rutgers.edu (A.J.); 2 Department of Chemistry and Chemical Processing Technologies, Cumra Vocational School, Selcuk University, 42130 Konya, Turkey

**Keywords:** gelatin, hydrogel microparticles, microgels, human mesenchymal stem cells (hMSCs), wound healing

## Abstract

The demand for innovative therapeutic interventions to expedite wound healing, particularly in vulnerable populations such as aging and diabetic patients, has prompted the exploration of novel strategies. Mesenchymal stem cell (MSC)-based therapy emerges as a promising avenue for treating acute and chronic wounds. However, its clinical application faces persistent challenges, notably the low survivability and limited retention time of engraftment in wound environments. Addressing this, a strategy to sustain the viability and functionality of human MSCs (hMSCs) in a graft-able format has been identified as crucial for advanced wound care. Hydrogel microparticles (HMPs) emerge as promising entities in the field of wound healing, showcasing versatile capabilities in delivering both cells and bioactive molecules/drugs. In this study, gelatin HMPs (GelMPs) were synthesized via an optimized mild processing method. GelMPs with distinct diameter sizes were sorted and characterized. The growth of hMSCs on GelMPs with various sizes was evaluated. The release of wound healing promoting factors from hMSCs cultured on different GelMPs were assessed using scratch wound assays and gene expression analysis. GelMPs with a size smaller than 100 microns supported better cell growth and cell migration compared to larger sizes (100 microns or 200 microns). While encapsulation of hMSCs in hydrogels has been a common route for delivering viable hMSCs, we hypothesized that hMSCs cultured on GelMPs are more robust than those encapsulated in hydrogels. To test this hypothesis, hMSCs were cultured on GelMPs or in the cross-linked methacrylated gelatin hydrogel (GelMA). Comparative analysis of growth and wound healing effects revealed that hMSCs cultured on GelMPs exhibited higher viability and released more wound healing activities in vitro. This observation highlights the potential of GelMPs, especially those with a size smaller than 100 microns, as a promising carrier for delivering hMSCs in wound healing applications, providing valuable insights for the optimization of advanced therapeutic strategies.

## 1. Introduction

Wound healing is an intricate process comprised of three distinct yet overlapping stages mediated by cell–cell and cell–matrix interactions [1,2]. During the initial phase of wound healing (inflammation phase), various types of white blood cells, such as neutrophils, macrophages, and T cells, are drawn to the injury site through chemical signals. Neutrophils eliminate bacteria and debris, while macrophages clear dead cells and generate growth factors, aiding healing. Activated T cells produce cytokines and growth factors, supporting the growth of other involved cells like fibroblasts and keratinocytes, when prompted by signals from cells like macrophages and dendritic cells. Once wound healing transitions into the second phase (proliferative phase), fibroblasts in the wound environment proliferate to produce extracellular matrix, particularly collagen, and release signals to initiate re-epithelialization [3]. Keratinocytes are vital in this phase, migrating and multiplying to cover the wound. Endothelial cells facilitate angiogenesis, forming new blood vessels crucial for providing nutrients and oxygen. Together, these cells collaborate to repair damaged tissues, transitioning collagen fibers from type III to type I, gradually enhancing the strength and flexibility of repaired tissues as epithelialization progresses [4,5,6]. The duration and quality of wound healing depend on many factors such as local cells or cells migrated to the injury area, growth factors, cytokines, and chemokines, each of which contributes to and regulates the overall wound healing process [7,8,9]. Deviations from the well-orchestrated process lead to delayed wound healing and even chronic wounds or fibrotic diseases [10]. 

Unhealed wounds not only negatively impact the quality of life of patients but present a significant burden to the healthcare system [11]. Based on a limited data set (Medicare beneficiaries in the United States in 2014), the costs associated with chronic nonhealing wounds ranged from USD 28.1 to 96.8 billon [12]. This high cost is expected to increase due to the rising number of diabetic and bariatric patients and an aging population [13,14]. 

The devastating effects on patients and alarming costs necessitate the development of more effective wound treatments. In recent years, many new therapeutic strategies have emerged to compliment the standard wound treatment care: TIME (tissue debridement, infection control, moisture balance, and edges of the wound) [10,15]. Among these strategies, cell therapy has gained traction [9,16]. Mesenchymal stem/stromal cells (MSCs) are the top candidate for cell therapy. MSCs are multipotent cells, which can differentiate into multiple cell lineages such as adipocytes, osteoblasts, chondrocytes, myocytes, and tenocytes [17,18,19]. Dental pulp, dermal tissues, adipose tissue, bone marrow, umbilical cord or Warton’s jelly are some of the tissue sources from which MSCs can be isolated [20,21,22]. The major reparative effect of these cells is attributed to paracrine signaling, which enhances the migration, proliferation, and survival of the cells involved in wound healing. MSCs release factors both directly and indirectly reduce inflammation and stimulate processes such as angiogenesis, re-epithelialization, and extracellular matrix (ECM) deposition [23,24,25]. Furthermore, clinical studies have demonstrated the safety of utilizing MSCs-based therapies, including in wound treatments [26,27]. While its potential is recognized, the use of MSCs in wound care still faces challenges. One obstacle is maintaining the bioactivity of MSCs in injured cutaneous tissue after their delivery [28,29]. Delivering MSCs directly to the wound through injection has been associated with issues such as low viability, transient retention, and overall poor efficacy. In recent years, methods have been developed to improve MSCs survival, focusing on the use of biocompatible scaffolds including natural or synthetic polymers and genetically engineered peptides [30,31,32]. The benefits of a scaffold in a wound environment include mimicking the natural ECM, providing mechanical support, and enhancing nutrient and oxygen transport to support the viability and functionality of MSCs [33].

Hydrogels constitute three-dimensional (3D) structures formed through the cross-linking of hydrophilic polymer networks. They exhibit a remarkable capability to absorb and preserve significant quantities of water and various biological fluids, making them a promising candidate for cell delivery [33]. Hydrogels can deliver MSCs safely to wound sites and protect them from an immune system attack [34]. The modified hydrogel microenvironment can support the proliferation of cells by regulating biophysical and biochemical properties, such as hydrogel–cell interactions, cell adhesion, microstructure, and degradability [35]. Collagen, natural ECM protein, or gelatin, the denatured form of collagen, can maintain cell adhesion patterns. Dong et al. reported that a gelatin-based hydrogel delivery mechanism enhanced the survival ratio of adipose-derived stem cells in diabetic wounds, and gelatin bulk hydrogel containing adipose-derived stem cells improved wound closure and neovascularization [36,37]. However, once applied on the wound site, the limited interface between bulk hydrogel and the wound causes low tissue infiltration and low rate of stem cells survival [38]. Therefore, alternative geometries or forms of hydrogel have been explored for efficient delivery of stem cells. Compared to bulk hydrogel, the geometry of hydrogel microparticles (HMPs), also known as microgels, provides a large surface area for cell growth and easy access to nutrients and cell-released factors [39,40,41]. Gelatin, one of the common biopolymers, presents advanced properties such as low antigenicity, good biodegradability, and biocompatibility in the physiological media [42]. It can be fabricated in microgel form, which can be employed alone or in combination with other biopolymers in manufacturing tissue engineering scaffolds [43], and can be degraded by the enzymes secreted from cells [44]. The proliferation and differentiation of various types of cells on gelatin HMPs have been reported previously. These cells include adipose-derived stem cells [38,45,46,47], MC3T3-E1 [48,49,50,51,52], bone-marrow-derived stem cells [53,54,55,56,57], preadipocytes [58], and cardiac progenitor cells [59]. 

This study aims to optimize the size of GelMPs as cell carriers for hMSCs in the context of wound healing. While the compatibility of gelatin HMPs (GelMPs) with cells is well established, the wound healing impact of human bone-marrow-derived stem cells (hMSCs) cultured on GelMPs remains unexplored, particularly in comparison with hMSCs encapsulated in gelatin hydrogels. To achieve this, GelMPs of various sizes were prepared and characterized, assessing parameters such as size, cross-linking degree, degradation rate, water content, and ATR-FTIR. The viability of hMSCs on GelMPs (hMSC/GelMPs) of different sizes was monitored for 14 days. The wound healing effect of factors released by hMSCs/GelMPs was evaluated using a scratch wound assay of human dermal fibroblasts (HDF). GelMPs with a size smaller than 100 microns (GelMPs < 100) supported better viability and wound healing activity compared to GelMPs with sizes between 100 and 200 microns or larger than 200 microns. Furthermore, the growth of hMSCs cultured on GelMPs < 100 was compared with hMSCs encapsulated in cross-linked methacrylated gelatin (GelMA) hydrogels. hMSCs grew on the surface of GelMPs and in GelMA gels with a preference on GelMPs. The wound healing activities of conditioned media from hMSCs cultured on GelMPs or in GelMA gels were tested using a scratch wound assay of human dermal fibroblasts (HDF) and the gene expression of wound healing factors such as platelet-derived growth factor (PDGF) in human dermal microvascular endothelial cells (HDMECs). Our results showed that the hMSCs cultured on GelMPs < 100 promoted the migration of HDF and stimulated the expression of PDGF in HDMECs as compared to hMSCs cultured in GelMA gels. We report here, for the first time, that GelMPs < 100 could serve as a promising cell carrier for hMSCs in wound care applications.

## 2. Results and Discussion

### 2.1. Synthesis of Gelatin Hydrogel Microparticles (GelMPs)

The water-in-oil emulsion technique is a widely used approach for producing gelatin microparticles [60,61]. After initial microparticle formation, the process includes cross-linking, washing, filtration, and lyophilization for prolonged storage [40]. The gentle processing conditions allow for the loading of proteins/drug molecules or cells during hydrogel microparticle formation [62]. Alternatively, diffusion-based loading can be achieved post-synthesis by incubating MPs with a concentrated protein solution [63]. In a typical water-in-oil emulsion process, chemical cross-linkers, commonly genipin or glutaraldehyde, are employed, eliminating the need for harsh cross-linking steps such as thermally initiated free radical polymerization [61,63]. In this study, glutaraldehyde was employed as a chemical cross-linker due to its recognition as the most widely used molecule owing to its cost-effectiveness, low toxicity at low concentrations, and efficacy in stabilizing collagenous materials. 

**Figure 1:**
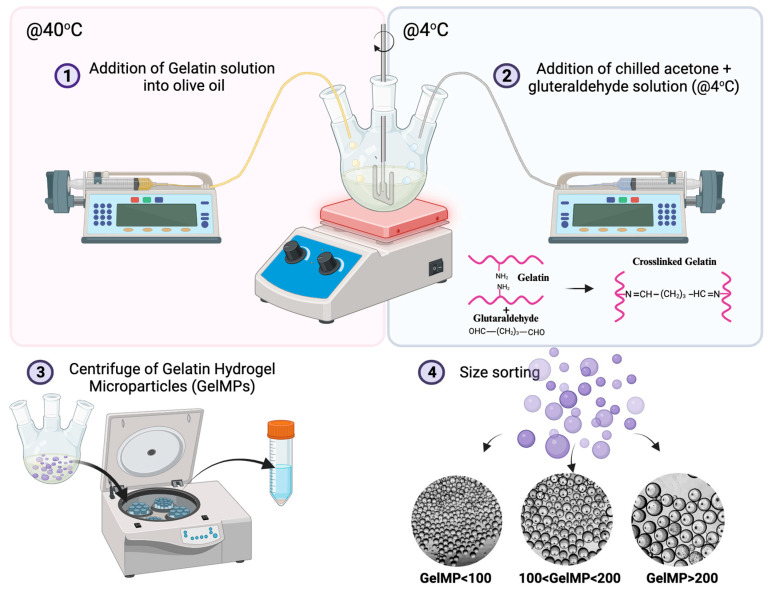
Schematic representation of gelatin hydrogel microparticle (GelMPs) production.

In our study, GelMPs were generated via a modified synthesis procedure utilizing the water-in-oil emulsion method to ensure distribution of water-soluble hydrogel precursors within the organic phase (Figure 1) [60]. Briefly, porcine gelatin type-A was dissolved in 10 mL of distilled water at 55 °C to create a clear solution. This solution was then added to preheated olive oil (40 °C) under constant stirring, resulting in a water-in-oil emulsion with a uniform distribution of GelMPs. The system underwent further processing, including mixing at 40 °C, cooling to 4 °C, cross-linking with glutaraldehyde, incubation, centrifugation, glycine treatment, and size-based sorting into three groups.

### 2.2. Characterization of Gelatin Hydrogel Microparticles (GelMPs) 

Polydispersity presents challenges, particularly in applications like controlled release or cell encapsulation, where regulating encapsulated drug quantity or cell numbers is difficult [41]. To overcome this, filtration was used for specific size selection while ensuring high-throughput particle production. In our study, GelMPs were separated into three size-specific groups using cell strainers. GelMPs with sizes smaller than 100 microns, between 100 microns and 200 microns, and larger than 200 microns were named as GelMP < 100, 100 < GelMP < 200, and GelMP > 200, respectively. 

**Figure 2:**
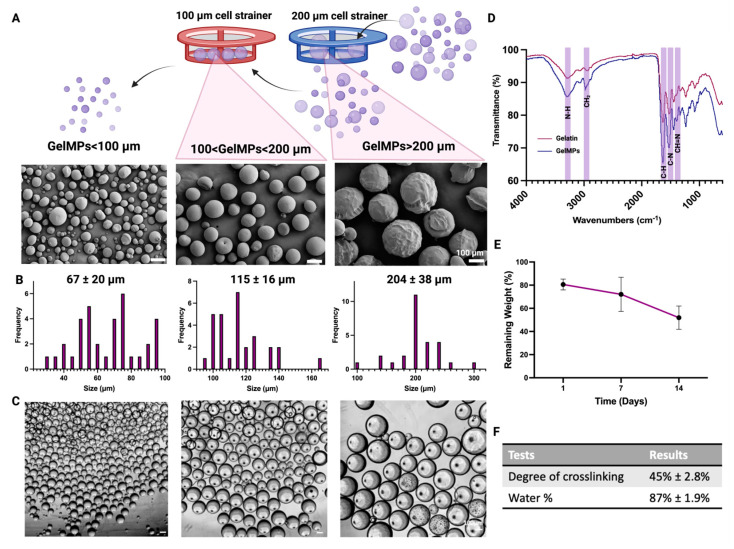
Size sorting and characterization of GelMPs. (**A**) Polydisperse GelMPs were sequentially size-sorted using 100 μm and 200 μm cell strainers, and sizes were assessed using FE-SEM micrographs of size-sorted GelMPs. (**B**) Size distribution of the GelMPs was analyzed using NIH Image J (Fiji for Mac OS X). (**C**) Optical images of size-sorted GelMPs taken with bright-field optical microscopy. (**D**) FT-IR analysis of gelatin and GelMP < 100. (**E**) in situ degradation of GelMP < 100. Data shown are mean ± SD (*n* = 4) and (**F**) degree of cross-linking, water %.

The morphology, the shape and size of GelMPs prepared as described in Section 4.1., was assessed by an optical microscope and quantified using NIH Image J (Fiji for Mac OS X). Figure 2A illustrates the general morphology of the microparticles, while Figure 2B showcases the particle histograms of each size group. The correlation between particle size and surface-to-volume ratio is widely acknowledged, with the surface-to-volume ratio increasing as the radius of spherical particles decreases [64]. The anticipated relationship suggests that GelMPs < 100 exhibit higher surface-to-volume ratio, thereby providing more surface area for the cell proliferation compared to larger sized GelMPs (Supplementary Material Table S1). The morphology of size-sorted GelMPs was analyzed using FE-SEM, revealing insightful findings on their shape and dimensions. As illustrated in Figure 2A, the micrographs provide an overview of the GelMPs, showcasing a spherical shape within a size range that aligns closely with the observations from optical images presented in Figure 2C. Additionally, the average particle sizes for each size group were determined to be 67 ± 20 μm for GelMP < 100, 115 ± 16 μm for 100 < GelMP < 200, and 204 ± 38 μm for GelMP > 200 (Figure 2B), respectively, following the size-sorting process.

Glutaraldehyde cross-linking occurs via interactions of the aldehyde groups in glutaraldehyde with the unprotonated amine groups of lysine and hydroxylysine and the amino groups of N-terminal amino acids [65,66]. To confirm the cross-linking of GelMPs, both cross-linked gelatin MPs and uncross-linked gelatin were analyzed using ATR-FTIR (Figure 2D). In the ATR-FTIR spectra of GelMPs, characteristic stretching vibrations of N-H bonds (due to the amide-A and amide-I groups of gelatin) were observed at 3295 cm^−1^ and 1633 cm^−1^, respectively [67]. The peak at 2940 cm^−1^ corresponds to the asymmetric stretching of CH_2_ groups in gelatin. Additionally, the peak at 1538 cm^−1^ is attributable to the vibration of amide-II, resulting from N-H stretching and C-N bending vibrations [67].

While the ATR-FTIR spectrum of gelatin microparticles shares some similarities with the ATR-FTIR spectrum of gelatin precursors, a distinct peak at 1450 cm^−1^ emerges, likely indicative of the aldimine linkage (CH=N) formed by glutaraldehyde-cross-linked GelMPs in the ATR-FTIR spectrum of GelMPs [68]. Furthermore, a comparison of the ATR-FTIR spectrum of gelatin precursors with that of GelMPs reveals changes in the band intensities of amino groups in the range of 3200–3400 cm^−1^, reflecting the cross-linking with glutaraldehyde [69]. Consequently, the overall ATR-FTIR results suggest that GelMPs are cross-linked by glutaraldehyde. 

The degree of cross-linking, defined as the quantity or fraction of reacted functional groups, was determined through a widely employed and reliable ninhydrin assay. This quantitative method involves the reaction of primary amines with ninhydrin, producing Ruhemann’s purple and yielding absorbance readings at 570 nm [70,71]. For the prepared GelMPs, the degree of cross-linking was determined to be 45 ± 2.8%, by utilizing a calibration curve of glycine (*n* = 8) (Supplementary Material Figure S1). The cross-linking degree of GelMPs is relatively comparable to that found in literature studies using glutaraldehyde as a cross-linker, primarily varying depending on the cross-linking time and concentration of the cross-linker [72,73]. The cross-linking degree can be fine-tuned by adjusting the concentration of the cross-linker and/or the polymerization duration, offering customizable physical and mechanical properties [61,74]. Unreacted functional groups in the gelatin backbone offer opportunities for additional functionalization, if desired, allowing modification or the creation of composites to enhance biocompatibility, stability, and bioactivity [66,75]. 

For optimal remodeling, the scaffold must degrade appropriately. Gelatin, a biodegradable natural polymer, undergoes enzymatic degradation by serine protease, collagenase, and metalloproteinases (MMPs) secreted by inflammatory cells [76] as well as by hydrolytic degradation through solvation and depolymerization of polymeric chains [77]. GelMPs’ stability was examined in PBS over 2 weeks to simulate in vitro cell culture conditions but avoid protein or lipid adsorption, revealing that the GelMPs lost 52% of their initial weight at the end of 2 weeks culturing (Figure 2E). The GelMPs demonstrated a retention of 88 ± 1.9% water at equilibrium following 24 h of incubation in PBS (Figure 2F). Tailored degradation and water content profiles are attainable by varying cross-linker concentrations [78], employing dual-cross-linking methods like enzymatic and ionic cross-linking [79], or forming composites with other materials like chitosan [80]. 

### 2.3. Growth of hMSCs on GelMPs In Vitro

Effective wound healing relies on a coordinated interaction among extracellular matrix proteins, growth factors, and cells. MSCs, being self-renewing multipotent stem cells, possess the ability to differentiate into various mesenchymal lineages, such as tendon, cartilage, and fat [23,81]. Beyond their multilineage differentiation potential, MSCs exhibit robust tissue-protective and -reparative activities via secreting growth factors and immune-modulators. The positive impact of exogenous MSCs on wound healing has been evident in various animal models [82] and reported clinical cases [83], demonstrating the successful application of cell-based therapy using MSCs in treating chronic wounds [7,81]. While systemic administration of hMSCs has proven effective in expediting wound healing in cutaneous wound models [84], delivering cells on a larger scale presents challenges in terms of cell engraftment and survival [81]. This challenge has spurred the exploration of local delivery methods through collaborative efforts between stem cell researchers and bioengineers.

**Figure 3:**
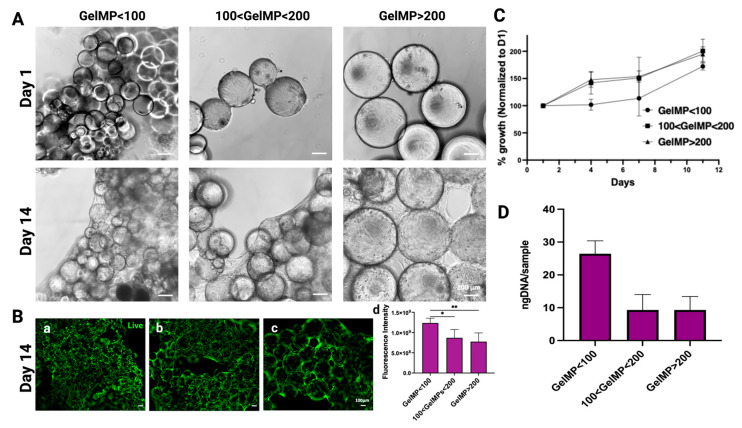
The growth of hMSCs on GelMPs of various sizes. (**A**) Phase contrast images of hMSCs on GelMP < 100, 100 < GelMP < 200, and GelMP > 200 from day 1 (upper panel) and day 14 (lower panel), scale bar = 100 µm. (**B**) Calcein AM staining of hMSCs on (**a**) GelMP < 100 (**b**) 100 < GelMP < 200 (**c**) GelMP > 200 on day 14, scale bar = 100 µm. (**C**) The viability of hMSCs on GelMPs was monitored using alamarBlue assay for 11 days. (**D**) DNA quantification of hMSCs on GelMPs on day 14. Data shown are mean ± SD (*n =* 4) * *p* < 0.05 and ** *p* < 0.01.

In our study, we explored the potential of GelMPs as an effective carrier for hMSC delivery and viability. Over a 14-day period, the growth of stem cells on various sizes of GelMPs was microscopically monitored. Over this period, microparticles displayed effective self-assembly with strong cell attachment, elongation resembling their natural environment, and formation of cell-to-cell connections, maintaining viability (Figure 3A). Live staining of hMSCs with CalceinAM on the GelMPs revealed overall cell viability, notably with higher fluorescence intensity observed for cells on GelMP < 100 (Figure 3(Ba)) when compared to cells on other sizes (Figure 3(Bb,Bc)). GelMP < 100 consistently exhibited a higher cell number based on fluorescence intensity readings (Figure 3(Bd)). Concurrently, the cell viability assay, alamarBlue, was conducted on days 1, 4, 7, and 13. The results revealed that hMSCs grow on GelMPs with the same trend in each of the size groups in terms of cell viability (Figure 3C), and this result was corroborated by DNA quantification using picogreen (Figure 3D). 

### 2.4. Effect of Conditioned Media from hMSCs Cultured on GelMPs of Varying Sizes on the Migration of Human Dermal Fibroblasts (HDF) 

**Figure 4:**
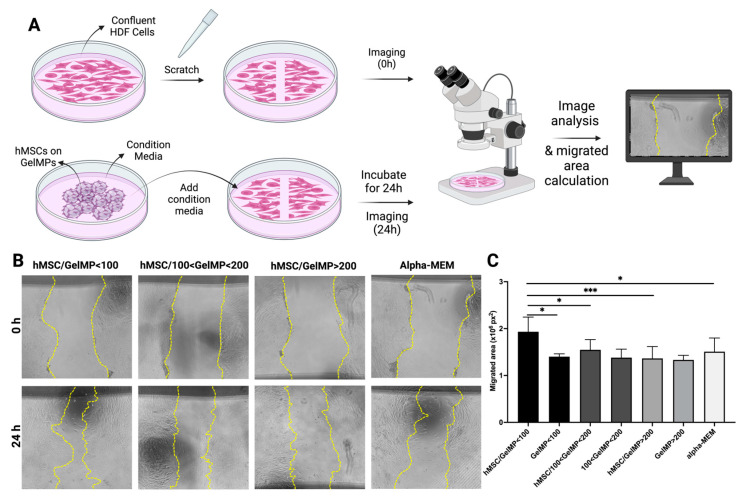
Migration of HDF in the presence of serum-free conditioned media collected from hMSCs cultured on GelMPs of varying sizes. (**A**) Schematic representation illustrating the wound scratch assay process. (**B**) Representative images of wound areas of HDF at 0 h (upper panel) and 24 h (lower panel). The wound edges were traced with yellow dotted lines. (**C**) Quantification of migrated area. The areas of wounds were measured using NIH Image J (Fiji for Mac OS X) software and the migrated area (px^2^) = Area_0h_ − Area_24h_. GelMP < 100, 100 < GelMP < 200, or GelMP > 200 were conditioned media collected from microparticles in the absence of hMSCs. Alpha-MEM is the complete medium (containing 10% fetal bovine serum) for culturing hMSCs. Data shown are mean ± SD (*n* = 8 for hMSCs on GelMPs and *n* = 4 for GelMPs alone). * *p* < 0.05 and *** *p* < 0.005.

It has been reported that the growth factors and cytokines secreted by hMSCs promote the migration of fibroblasts, thereby contributing to a positive enhancement of the wound healing process [85,86]. The in vitro scratch assay provides a simple, cost-effective, and direct approach to investigate cell migration in vitro, aiming to mimic the in vivo cell migration process. This method involves generating an artificial gap or “scratch” on a monolayer of confluent cells, prompting the cells at the edge of the gap to migrate towards the opening by forming new cell–cell contacts. The assessment of the treatment’s efficacy on the cells is determined by calculating the areas of migration, derived from initial images and subsequent images captured at regular intervals (Figure 4A) [87]. To evaluate whether hMSCs cultured on GelMPs of varying sizes exhibit distinct effects on wound healing, we compared the impact of serum-free conditioned media from hMSCs cultured on GelMP < 100, 100 < GelMP < 200, or GelMP > 200 on the migration of HDF in a scratch wound assay (Figure 4B). 

Among the three sizes compared, the conditioned media collected from hMSCs cultured on GelMP < 100 demonstrated significantly higher activity in promoting the migration of HDF in this scratch wound assay (Figure 4C). While the conditioned media from hMSC/100 < GelMP < 200 appeared to exhibit slightly better activity than that of hMSC/GelMP > 200, no statistically significant difference was observed. The enhanced migration-promoting activity of hMSC/GelMP < 100 may be attributed to the more robust growth of hMSCs on GelMP < 100 providing more surface area for enhanced cell proliferation (see Figure 3). This, in turn, leads to the increased secretion of growth factors that positively regulate HDF migration. Based on the in vitro results discussed with the assessments of cell proliferation on GelMPs and a scratch assay simulating wound healing conditions, GelMP < 100 media were selected for further subsequent in vitro studies.

### 2.5. Comparison of hMSCs Growth on GelMP < 100 µm versus Encapsulated in GelMA

Utilizing bulk hydrogels for delivering cells to the target area is a widely employed method in wound dressing [88,89,90], with GelMA emerging as a prominent derivative due to its customizable chemical structure, biocompatibility, biodegradability, and capacity to facilitate the attachment and growth of diverse cell types [42,91,92]. In our study, hMSCs encapsulated in GelMA hydrogels served as the control group in comparison to hMSCs seeded on HMPs. The synthesis, following the protocol outlined in [93,94,95], and characterization of GelMA are detailed in the Supplementary Information, along with Supplementary Figures S2 and S3.

**Figure 5:**
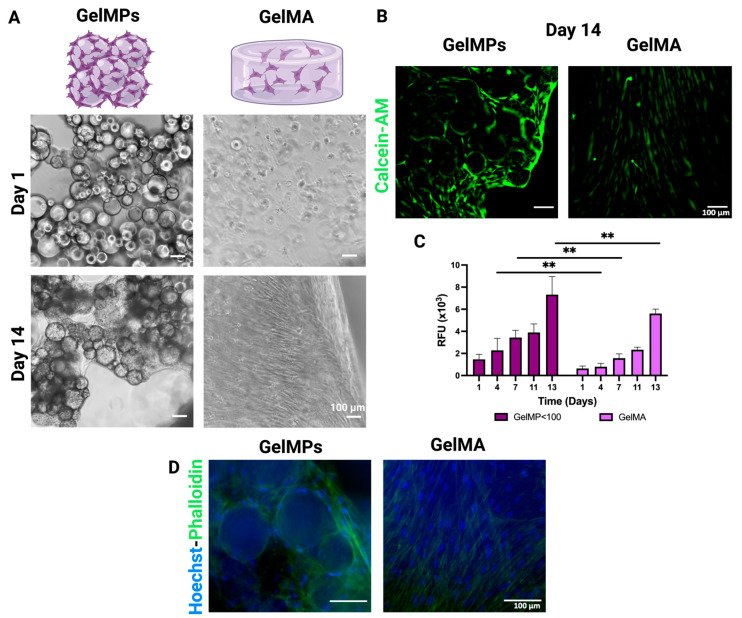
In vitro studies of hMSCs on GelMPs versus embedded in GelMA for 14 days. (**A**) Phase contrast images captured on day 1 and day 14. (**B**) Calcein AM staining of hMSCs on GelMPs and in GelMA on day 14. (**C**) The viability of hMSCs on GelMPs versus in GelMA was monitored using alamarBlue assay for 13 days. Data shown are mean ± SD (*n* = 4). (**D**) Phallodin–Alexa 488 (actin cytoskeleton, green)- and Hoechst (nuclei, blue)- stained hMSCs on GelMPs versus embedded in GelMA on day 14, scale bar = 100 µm. ** *p* < 0.01.

The growth curve of hMSCs on GelMPs versus encapsulated in GelMA hydrogels exhibited similar trends in terms of cell viability. Figure 5A shows representative images of hMSCs on GelMPs and in GelMA from day 1 and day 14, revealing the cell attachments and cell–cell connections around the GelMPs and within the GelMA hydrogels. The results suggest the biocompatibility of both systems, supporting cell growth over the 2-week culture period. Live staining of hMSC on GelMPs presented overall cell viability, shown with higher fluorescence intensity detected for cells on GelMP < 100 μm compared to cells in GelMA (Figure 5B), corroborating the relative fluorescence units (RFU) reading of alamarBlue assay (Figure 5C). Phalloidin-Hoechst staining was performed on day 14 in order to analyze the morphology of actin filaments. hMSCs on GelMPs showed strong cytoskeletal actin staining surrounding and in between the MPs, while hMSCs in GelMA showed aligned organization of cell cytoskeletal structures after 14 days. 

### 2.6. Effect of Conditioned Media from hMSCs Cultured on GelMP < 100 µm or in GelMA on the Migration of HDF 

**Figure 6:**
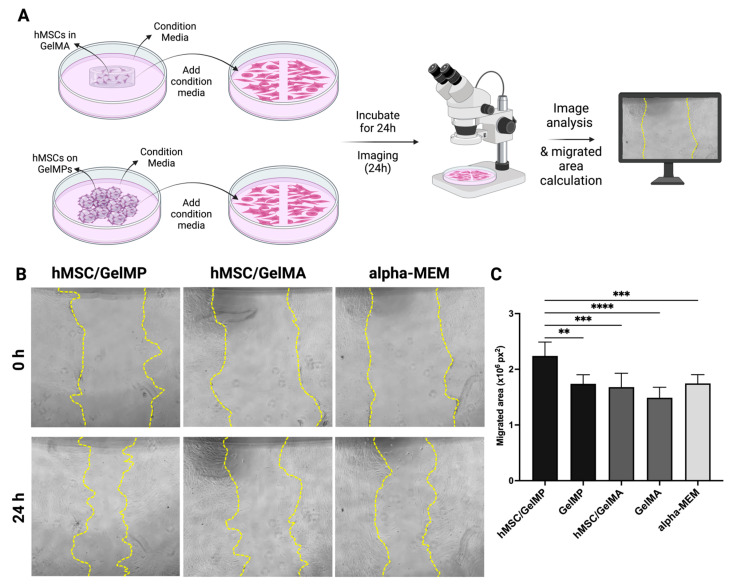
Migration of HDF in the presence of serum-free conditioned media collected from hMSCs cultured on GelMPs or from hMSCs cultured in GelMA. (**A**) Schematic representation illustrating the process. (**B**) Representative images of wound areas of HDF at 0 h (upper panel) and 24 h (lower panel). The wound edges were traced with yellow dotted lines. (**C**) Quantification of migrated area. The areas of wounds were measured using NIH Image J (Fiji for Mac OS X) software and the migrated area (px^2^) = Area_0h_ − Area_24h_. GelMP or GelMA were conditioned media collected from microparticles or GelMA gels in the absence of hMSCs. Alpha-MEM is the complete medium (containing 10% fetal bovine serum) for culturing hMSCs. Data shown are mean ± SD (*n* = 8 for hMSCs with GelMPs or GelMA and *n* = 4 for GelMPs or GelMA alone). ** *p* ≤ 0.01, *** *p* < 0.005, and **** *p* < 0.001.

To evaluate whether hMSCs cultured on GelMP < 100 µm or encapsulated in GelMA have comparable wound healing activity, the effects of conditioned media collected from hMSCs cultured on GelMPs (hMSC/GelMP) or from hMSCs encapsulated in GelMA (hMSC/GelMA) on the migration of HDF were tested in a scratch wound assay (Figure 6A,B). 

As shown in Figure 6B, the serum-free conditioned media collected from hMSCs cultured on GelMPs showed significant stronger stimulatory effect on the migration of HDF than the conditioned media from hMSCs cultured in GelMA. While hMSCs showed strong viability in GelMA, the secretion and release of migration-promoting factors of hMSCs are likely less effective than cells cultured on GelMPs. 

### 2.7. The Effect of Conditioned Media on the Gene Expression of Wound Healing Promoting Factor: PDGF 

Wound healing is an essential physiological process consisting of a sequence of molecular and cellular events which occur immediately after the onset of an injury to restore damaged tissue. The process encompasses several stages, namely the inflammatory reaction, cell proliferation, synthesis of extracellular matrix components, and the subsequent remodeling phase [2,96]. These phases exhibit considerable overlap, with the initial crucial step being hemostasis, occurring within seconds or minutes of the wound. Platelets play a pivotal role by forming a blood clot to prevent blood loss and microbial entry, releasing essential signaling molecules such as platelet-derived growth factor (PDGF), transforming growth factor-beta (TGF-b), epidermal growth factor (EGF), and fibroblast growth factor (FGF), crucial for subsequent healing phases [2].

**Figure 7:**
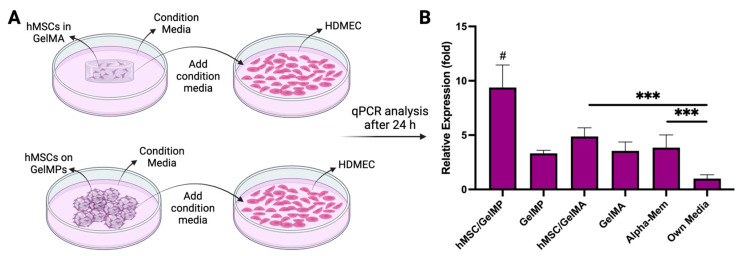
qPCR analysis of HDMECs following treatment with various media (**A**) The schematic outlines the process of HDMEC treatment with conditioned media (starvation media) derived from cultures of hMSCs on GelMPs and hMSCs embedded in GelMA hydrogels. (**B**) Relative expression of PDGFB gene in HDMECs in the presence of conditioned media. Data shown are mean ± SD (*n =* 4). *** *p* < 0.005 and # indicates a statistically significant difference against all other conditions.

PDGF-BB (made of two B subunits) plays a pivotal role in tissue healing by participating in inflammatory responses, neovascularization, chemotaxis, proliferation stimulation, and matrix production [97,98,99]. It is secreted by platelets immediately after wounding to initiate multiple wound healing signaling pathways. As the healing progresses, other cell types in the wound environment, such as endothelial cells, begin to secrete PDGF-BB [97]. In this investigation, the immunomodulatory impact of hMSCs GelMPs or in GelMA was evaluated by comparing their effects on the gene expression of PDGFB in human dermal microvascular endothelial cells (HDMECs) (Figure 7). To eliminate the confounding influence of serum, hMSCs cultured on GelMPs and encapsulated in GelMA were incubated in serum-free media for 24 h (Figure 7A). Subsequently, the conditioned media collected from this culture was used to incubate HDMECs cells for 24 h. HDMECs were also subjected to incubation in serum-containing complete hMSC media and their own media (endothelial cell growth medium) as control conditions. Quantitative PCR (qPCR) results, normalized to cells incubated in their own media, revealed that the media collected from hMSCs on GelMPs significantly promoted the expression of PDGFB in HDMECs compared to other groups, highlighting the wound healing activity of hMSCs on their carriers, GelMPs (Figure 7B). 

In this study, we assessed the expression of PDGFB, a key regulator of wound healing. However, to validate the impact of hMSCs on GelMPs in wound healing, it is essential to conduct additional in vitro assays, including angiogenic assays and the dynamic expression analysis of other wound healing modulators. Moreover, conducting wound healing studies in an animal model will provide further insights and confirmation of these effects.

## 3. Conclusions

In conclusion, the culmination of the various analyses and experiments presented throughout our exploration of wound healing underscores the pivotal role of hMSCs and specialized carriers in fostering effective tissue regeneration. The scratch assays and qPCR results collectively illuminate the significant impact of hMSCs on wound healing processes. Particularly noteworthy is the role of GelMP < 100 µm as a promising carrier, offering both versatility and potential for further functionalization owing to the presence of functional groups within GelMPs, combined with mild processing conditions. Therefore, in future studies, loading with bioactive molecules such as growth factors and cytokines to elevate stem cell activity, promote proliferation, and augment the secretion of vital signaling molecules would contribute to a more efficient and accelerated wound healing response. A crucial aspect of this exploration involves examining the conditioned media’s content through ELISA, providing a comprehensive understanding of how GelMPs influence the secretion of growth factors and signaling molecules, thereby facilitating effective microparticle content design. The findings presented here contribute to advancing our understanding of hMSC-mediated wound healing activities and provide a foundation for future developments in the field of regenerative medicine.

## 4. Materials and Methods 

### 4.1. Synthesis of Gelatin Hydrogel Microparticles (GelMPs)

Gelatin microspheres were synthesized by the water-in-oil emulsion modified method (Figure 1) [100,101,102,103]. Briefly, 2 g of gelatin (type A, porcine skin, 300 Bloom, Sigma-Aldrich, MO, USA) was dissolved in 10 mL of distilled water and stirred at 55 °C until dissolved. In a 250 mL round-bottomed flask, olive oil (VWR International, Radnor, PA, USA) was preheated to 40 °C in a water bath with an automatic overhead stirrer set-up. The dissolved gelatin was added to the preheated olive oil drop by drop, with stirring at 500 rpm. The emulsion solution was mixed at 40 °C for 30 min. Then, the reaction temperature was decreased to 4 °C by immersing the reaction flask in an ice bath and kept at that temperature for 30 min. The solution of 20 mL of chilled acetone (Sigma-Aldrich, St. Louis, MO, USA) and 0.2 mL of 25% glutaraldehyde (Sigma-Aldrich, St. Louis, MO, USA) was slowly added to the reaction flask and incubated for 1 h, with mixing at 4 °C. The emulsion was centrifugated at 3400 rpm at 5 °C for 5 min, and the supernatant was removed. The obtained GelMPs were washed with cold acetone to remove the olive oil that remained on the surface of microspheres. After the acetone wash, GelMPs were treated with 20 mL of 10 mM glycine (Fischer Scientific, Hampton, NH, USA) solution for 30 min on a rotator. The GelMPs were washed with water and subsequently sorted using specified filters (100 μm—Corning Inc., Corning, NY, USA; 200 μm—pluriSelect Life Science, Leipzig, Germany) into three different sizes: GelMP < 100 μm, 100 < GelMP < 200 μm, and GelMP > 200 μm. The success of the size-sorting process was verified using a Zeiss Sigma field emission scanning microscope (FESEM, Carl Zeiss, Oberkockhen, Germany) operating at an accelerating voltage of 3 kV and a working distance of 10.4 mm. Additionally, an optical microscope (Echo Revolve, San Diego, CA, USA) was employed for further confirmation. All groups were lyophilized and then stored at 4 °C for further analysis and use. 

### 4.2. Determination of Degree of Cross-Linking in GelMPs

Ninhydrin assay [71] was studied to calculate the percentage of free amino groups remaining in the GelMPs and GelMA after cross-linking processes. A ninhydrin (Sigma-Aldrich, St. Louis, MO, USA) solution was prepared right before performing the assay by dissolving 0.2 g of ninhydrin and 0.03 g of hydrindantin (Sigma-Aldrich, St. Louis, MO, USA) in 7.5 mL of dimethyl sulfoxide (DMSO, Sigma-Aldrich, St. Louis, MO, USA). In a separate flask, lithium acetate buffer was prepared by dissolving 8.16 g of lithium acetate (Sigma-Aldrich, St. Louis, MO, USA) in 12.0 mL of distilled water, then pH of the solution was set to 5.2, adding glacial acetic acid (Sigma-Aldrich, St. Louis, MO, USA), and the last volume was completed to 20.0 mL with distilled water. To prepare a working solution, 2.5 mL of lithium acetate buffer was added into the solution containing ninhydrin and hydrindantin in DMSO. For the assay, 2.5 mg of uncross-linked gelatin, GelMA or GelMPs was dissolved in 0.5 mL of distilled water in an Eppendorf tube, and 0.5 mL of the abovementioned working solution was added to each sample. The color of the samples in the tubes changed to a purple-blue color. The tubes were promptly capped, vortexed, and inserted in a heater set to 100 °C for 20 min. Then, the tubes were cooled down to room temperature, and 0.1 mL of the sample was mixed with 50% of isopropanol (Thermo Fisher Scientific, Waltham, MA, USA) and vortexed. The absorbance of each solution was measured at 570 nm using a TECAN Spark^®^ 10 M Plate reader (TECAN, Mannedorf, Switzerland), following the manufacturer’s instructions. The calibration curve was drawn by using glycine (Fisher Scientific, Hampton, NH, USA) within the concentration range of 160–0 μg/mL (*n =* 8). The cross-linking degree was determined according to Equation (1).

(1) $$CD\%=\frac{\left[NH_{2}gelatin-NH_{2}crosslinked\right]}{NH_{2}gelatin}\;\times 100$$

### 4.3. ATR-FTIR Analysis

The Thermo Scientific Nicolet iS10 ( Thermo Fisher Scientific, Madison, WI, USA) was utilized to analyze the lyophilized GelMPs, obtaining Attenuated Total Reflectance-Fourier Transform Infrared (ATR-FTIR) spectra for the samples.

### 4.4. Determination of Swelling Capacity of GelMPs

Lyophilized GelMPs were weighted (***W*_0_**) in Eppendorf tubes (*n =* 4) and sterilized under UV light for 30 min. The GMSs were incubated in 1.0 mL of PBS (Sigma-Aldrich, St. Louis, MO, USA) on a continuous shaker at 37 °C with 5% CO_2_ and 95% humidity for 24 h. After 24 h of shaking, the GelMPs were separated by centrifugation at 3400 rpm and washed with distilled water three times to eliminate any residue. Supernatant on GelMPs was removed gently and wet weighs (***W_w_***) were measured. The water content absorbed by GelMPs was determined from the following Equation (2):

(2) $$Water\;content\;\left(\%\right)=\frac{\left[W_{w}-W_{0}\right]}{W_{w}}\;\times 100$$

### 4.5. In Situ Degradation of GelMPs in PBS

Lyophilized GelMPs were weighted (***W*_0_**) in Eppendorf tubes (*n =* 4) and sterilized under UV light for 30 min. The GelMPs were then incubated in PBS (pH = 7.4) on a continuous shaker at 37 °C with 5% CO_2_ and 95% humidity for 14 days. At specified time points (days 1, 7, and 14), the remaining microparticles were centrifuged, washed with water to remove any residue, freeze-dried and weighed again (***W_x_***). The remaining weight (%) was determined according to equation described below:

(3) $$Remaining\;Weight\;\left(\%\right)=\frac{W_{x}}{W_{0}}\;\times 100$$

### 4.6. Culturing of hMSCs on GelMPs or in GelMA

GelMPs were sterilized under UV for 30 min before using and dispersed in Alpha-MEM (Gibco, Waltham, MA, USA) complete medium containing 10% FBS (Heat Inactivated, CPS Serum, Parkville, MO, USA) and 0.05% gentamicin (Sigma Aldrich, 50 mg/mL in deionized water) and mixed on a rotator for 2 h. For cell proliferation experiments, GelMPs were transferred to cell-repellent 48-well plates (Greiner Bio-One, Kremsmünster, Austria) at 0.5 mg per well, and human bone-marrow-derived mesenchymal stem cells (hMSC) (passage 4–5) (Texas A&M, 8011L, TX, USA) at a concentration of 1 × 10^4^ cells/well in alpha-MEM complete media was subsequently seeded onto the microspheres. The plates were placed on an orbital shaker (Benchmark Scientific, Sayreville, NJ, USA) in an incubator at 37 °C with 5% CO_2_ and 95% humidity. Growth media were changed every three days. 

The cell-laden GelMA hydrogels were molded on polydimethylsiloxane (PDMS) (Slygard 184, Dow Corning, Midland, MI, USA) templates. In brief, PDMS prepolymer and catalyst were mixed, subsequently poured into glass Petri dishes, and heated at 70 °C for a duration of 3 h. Then, the resulting PDMS film was punched to generate small holes of 8 mm and sterilized under UV light for 30 min. A GelMA solution of 10% *w*/*v* was made in complete media, along with 0.05 % (*w*/*v*) LAP photoinitiator (Lithium phenyl-2,4,6-trimethylbenzoylphosphinate) (Sigma-Aldrich, St. Louis, MO, USA) (Supplementary Material Figure S4). The GelMA solution was mixed with hMSCs (passage 4–5) at a concentration of 3.30 × 10^5^ cells/mL; subsequently, this solution was pipetted into PDMS molds (30 μL, containing 1 × 10^4^ cells) and cross-linked under a light source of 405 nm (Sovol, Shenzhen, China) at 20 mW cm^−2^ and at 13 cm for 40 s. The transparent hydrogels were transferred to cell-repellent 48-well plates, washed with alpha-MEM complete media, and incubated in the same media. The plates were positioned on an orbital shaker in an incubator at 37 °C with 5% CO_2_ and 95% humidity. Growth media were changed every three days.

### 4.7. AlamarBlue Assay

Following the seeding of 1 × 10^4^ cells on GelMPs per well and in GelMA per hydrogel, the adhesion and proliferation of hMSCs on GelMPs or within GelMA in alpha-MEM complete media were monitored. At the specific day points, alamarBlue assay was performed. In brief, growth media were aspirated, and subsequently, 0.2 mL/well of alamarBlue solution (complete growth medium + 10% alamarBlue reagent) (Bio-Rad Laboratories, Philadelphia, PA, USA) was put in each well and incubated at 37 °C for 30 min. After incubation, 0.1 mL of supernatant was transferred to a 96-well plate, then fluorescent intensity was examined using a multimode microplate reader at excitation/emission equal to 540 nm/590 nm. Fluorescent intensity was given in arbitrary units (AU). 

To continue the proliferation of cells on GelMPs or in GelMA, cells were treated with 0.5 mL/well of PBS once after alamarBlue assay. The samples were taken into alpha-MEM complete media for another 2 h of culturing in an incubator. Then, the complete media were aspirated, and 0.4 mL/well of fresh medium was introduced to each well, which was then cultured for the upcoming time point. AlamarBlue assay was performed to measure the cell viability at a specific time point. The results were evaluated according to following equation:

(4) $$Viability\;\left(\%\right)=\frac{FL_{\left(day\;T\right)}}{FL_{\left(day\;1\right)}}\;\times 100$$

### 4.8. Live Staining

Live staining was done after 14 days incubation of hMSC on GelMPs/in GelMA using Calcein-AM. Briefly, cells were stained with Calcein-AM (1 μM in PBS, Corning Inc., Corning, NY, USA) for 30 min at room temperature, washed with PBS and visualized using fluorescence microscope (Echo Revolve, San Diego, CA, USA). 

### 4.9. DNA Quantification of hMSC

At the end of hMSC culturing on GelMPs or in GelMA, 0.2 mL/well of RNA lysis buffer (Promega, Madison, WI, USA) was added on cells and frozen down at −80 °C. Then, the samples were thawed, and DNA was quantified using the Helixyte Green dsDNA Assay Kit (AAT Bioquest, Pleasanton, CA, USA).

### 4.10. Fluorescence Staining of hMSC-Laden GelMPs or GelMA

Immunofluorescent staining was performed after a 14-day culture of hMSCs on GelMPs or within GelMA. Briefly, cells were fixed with 4% paraformaldehyde (Thermo Fisher Scientific, USA) for 30 min and permeabilized in 0.5% Triton X100 in PBS for 2 h. The permeabilized samples were treated with Alexa Fluor^TM^ 488 Phalloidin (Ref#A12379, Life Technology, Carlsbad, CA, USA) at 1:40 dilution and Hoechst dye 33258, 20 mM in water (Cat #83219, AnaSpec Inc., Fremont, CA, USA) at 1:500 dilution for 30 min at 37 °C. After staining, samples were washed with PBS twice and imaged under a fluorescent microscope.

### 4.11. Conditioned Media for Migration Assay

Human bone-marrow mesenchymal stem cells (hMSC) were cultured on GelMPs with various sizes or within GelMA constructs in alpha-MEM complete medium for 14 days. To prepare conditioned media for the cell migration assay, alpha-MEM complete medium was removed from each culture. Subsequently, 0.45 mL/well of alpha-MEM base medium (without FBS) was added to each well, including those with cell-free gelatin microparticles or GelMA constructs as controls. The cultures were incubated at 37 °C with 5% CO_2_ and 95% humidity for 24 h. The supernatants (conditioned media) were collected from each well and immediately used in the migration assay. 

### 4.12. Scratch Wound Migration Assay

For the scratch wound migration assay, human dermal fibroblasts (passage 5–6) (ATCC, Manassas, VA, USA) at a density of 6 × 10^4^ cells per well were seeded onto tissue-culture-treated polystyrene 48-well plates and cultured in DMEM complete medium containing 10% FBS) at 37 °C with 5% CO_2_ and 95% humidity for 2 days. Scratch wounds were made on a confluent monolayer using a sterile 1 mL pipette tip. After removal of the medium from each well and rinsing with 0.4 mL/well of 1 × PBS, 0.2 mL/well of conditioned medium (collected as described above) was added to the wounds. Images of the wound areas were captured at 0 h, with two areas marked and monitored for each well. The plates were incubated at 37 °C for 24 h, and the exact same wound areas (with marker references) were imaged again at 24 h. Wound areas were measured using NIH Image J (Fiji for Mac OS X) software in arbitrary units (square pixels, px^2^). The migrated area was calculated as follows:

(5) $$Migrated\;area=Area_{0h}-Area_{24h}$$

### 4.13. qPCR Analysis 

HDMECs (passage 5–6) were seeded at a density of 20 × 10^4^ cells/well in 48-well plates. After the cells reached 80% confluency, the conditioned media from MSCs on GelMPs and encapsulated in GelMA hydrogels were added to HDMECs and incubated in the tissue culture incubator for 24 h. Complete alpha-MEM media and the own media of the HDMEC cells were used as controls. After 24 h, the medium was removed from each well. Cells were rinsed once with PBS and then were lysed with 0.2 mL/well RNA Lysis buffer (Promega, Durham, NC, USA). The RNA lysates were stored at −80 °C if not used immediately. 

The quantification of the relative expression of wound healing promoting factors was performed in HDMEC by qPCR, as previously described [104]. Briefly, total RNA from these lysates was purified using the SV 96 Total RNA Isolation System (Promega, Madison, WI, USA). RNA concentration and purity were measured using a TECAN Spark Nano plate (TECAN, Männedorf, Switzerland). cDNA preparation and qPCR were performed on a Roche Lightcycler 480 device following a standard procedure. Primers used in qPCR analysis were listed in Table 1. Every testing condition has 3–4 biological repeat samples, and each sample was run in duplicate. After the run was completed, a second derivative analysis was performed using the raw data to determine the mean Cp (Crossing point-PCR-cycle) for each sample. mRNA expression relative to GAPDH was determined by Pfaffl analysis:

(6) $$Relative\;gene\;expression=\frac{2^{\Delta Cp\;target}}{2^{\Delta Cp}\;reference}$$

in which: Δ***Cp*** = mean ***Cp*** of sample—mean ***Cp*** of the cells incubated in their own media.

### 4.14. Statistical Analysis

For each experiment, at least 3 samples (n ≥ 3) were used as biological repeats, and data are presented as mean ± standard deviation. Statistical analysis was performed as described [105]. One-way ANOVA with a Tukey’s multiple comparisons test was performed to determine statistical significance using GraphPad Prism version 10.1.1 (November 2023) for all quantitative data. Differences were considered significant at a *p* value of <0.05.

## Figures and Tables

**Table 1:** Primers (Qiagen, Germantown, MD, USA) and corresponding catalog numbers employed in qPCR analysis.

Primers	ID
Quanti-tect GAPDH	QT01192646
Quanti-tect PDGFB	QT00001260

## Data Availability

All data and materials are available on request from the corresponding author. The data are not publicly available due to ongoing researches using a part of the data.

## Supplementary Materials

The following supporting information can be downloaded at: https://www.mdpi.com/article/10.3390/gels10020097/s1, Figure S1: Calibration curve for Ninhydrin assay drawn by using glycine (Fisher Scientific, Hampton, NH, USA) within the concentration range of 160–0 μg/mL (n = 8); Figure S2: Schematic representation of methacrylated gelatin (GelMA) synthesis; Figure S3: ^1^H-NMR Spectra of GelMA and Gelatin. The upper-left panel depicts the corresponding chemical bonds; Figure S4: Schematic illustration detailing the fabrication process of GelMA hydrogels laden with hMSCs; Table S1. Comparison of the total surface area, total volume, and surface area-to-volume ratio of GelMPs samples.
