# Supplementary material for: Human Mesenchymal Stem Cells on Size-Sorted Gelatin Hydrogel Microparticles Show Enhanced In Vitro Wound Healing Activities
Source: Gels. 2024 Jan 26;10(2):97. doi: 10.3390/gels10020097 (PMC10887759; doi:10.3390/gels10020097)
Supplement: Supplementary file 1 [file gels-10-00097-s001.zip › gels-2814623-supplementary.pdf]

Article- Supplementary Information

Human Mesenchymal Stem Cells on Size-sorted Gelatin Hydrogel Microparticles Show Enhanced Wound Healing Activities

Derya Ozhava [1,2+], Cemile Bektas [1+], Kathleen Lee [1], Anisha Jackson [1] and Yong Mao [1*]

[1] Laboratory for Biomaterials Research, Department of Chemistry and Chemical Biology, Rutgers University, 145 Bevier Rd., Piscataway, NJ, 08854, USA
[2] Department of Chemistry and Chemical Processing Technologies, Cumra Vocational School, Selcuk University, 42130, Konya, Turkey
[+] Contributed equally
* Correspondence: Correspondence: Yong Mao, ORCID: 0000-0001-9268-4523 Tel.: (848) 445-9618, maoy@chem.rutgers.edu

1- Calibration Curve for Ninhydrin Assay

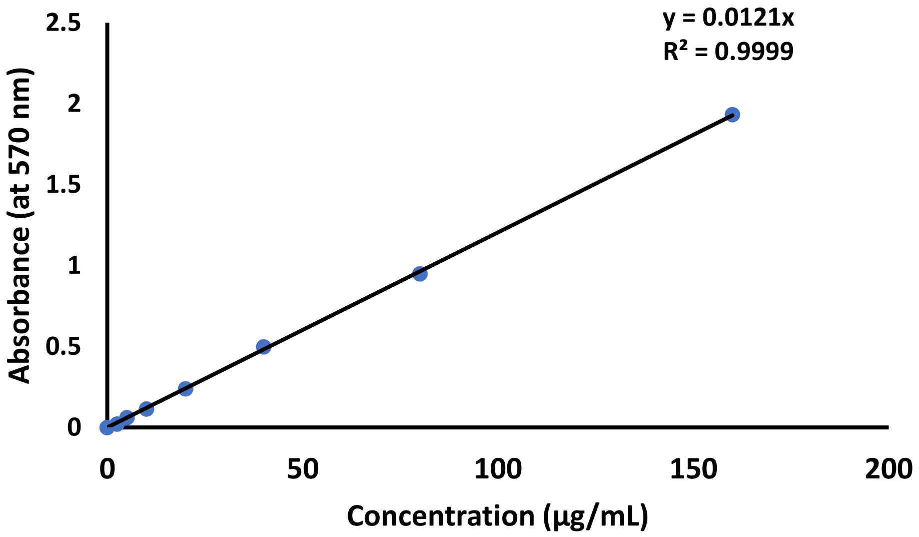

Figure S1: Calibration curve for Ninhydrin assay drawn by using glycine (Fisher Scientific, Hampton, NH, USA) within the concentration range of 160 – 0 µg/mL (n = 8).

2- Synthesis of Methacrylated Gelatin

To produce methacrylated gelatin (GelMA), the typical procedure previously published has been followed (Figure S2) [93]. Initially, 10% (w/v) gelatin solution was prepared in 100 mL of 0.25 M carbonate-bicarbonate buffer (Sigma-Aldrich, St. Louis, MO, USA) at 55℃, followed by adjusting the pH of the solution to 9.4. Subsequently, 0.938 mL of methacrylic anhydride (Sigma-Aldrich, St. Louis, MO, USA) was introduced to the reaction solution under continuous stirring at 500 rpm. After 1 hour of mixing at 55 ℃, the pH was adjusted to 7.4 to halt the reaction. The resulting solution was dialyzed against diH_2O at 37°C for 2 days, followed by filtration and lyophilization process. The degree of substitution was determined by performing 1H-NMR analysis and ninhydrin assay on both gelatin and GelMA.

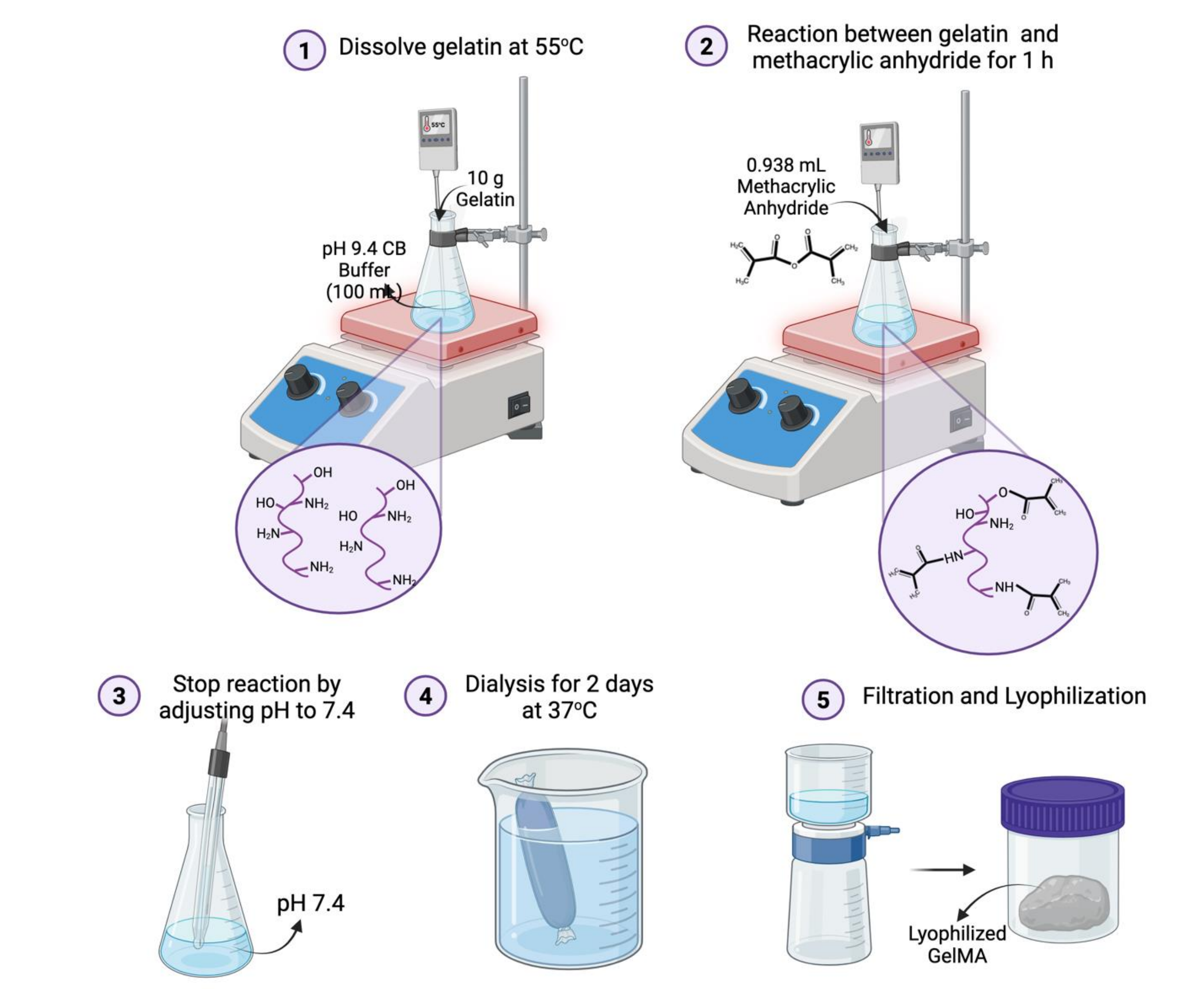

Figure S2: Schematic representation of methacrylated gelatin (GelMA) synthesis.

3- Calculation of Degree of Substitution

Degree of substitution was calculated using NMR (SI Figure 3) and ninhydrin assay.

^{1}H-NMR spectra were obtained as reported before [94]. Briefly, NMR The determination of the degree of substitution (DS) involved analyzing 1H-NMR spectra of both gelatin and GelMA to quantify methacrylate groups in GelMA, utilizing a Bruker DPX 500 spectrometer. 30 mg of GelMA and gelatin were individually

dissolved in 1 mL of deuterium oxide at 40 ◦C, and H-NMR spectra were acquired at the same temperature. Following phase and baseline corrections, the chemical shift scale was aligned to the phenylalanine signal (6.9–7.5 ppm). Normalization of the NMR spectra to the phenylalanine signal, proportional to the gelatin concentration, was performed. DS was calculated by integrating the areas of lysine methylene signals (2.8–2.95 ppm) of GelMA and gelatin using the provided equation.

$$Degree\ of\ Substitution\ (DS,\%) = \left(1 - \frac{Peak\ area\ (Lysine\ methylene\ of\ GelMA)}{Peak\ area\ (Lysine\ methylene\ of\ non-modified\ gelatin)}\right) x100$$

GelMA synthesis employed the recently introduced one-pot GelMA synthesis protocol (SI Figure 2) [93], offering advantages such as a brief reaction and purification period, consistent batch-to-batch outcomes, and precise control [93,95]. DS was determined to be approximately 100% through analysis of the NMR spectra as detailed above and 92% ± 1.4% using the ninhydrin assay.

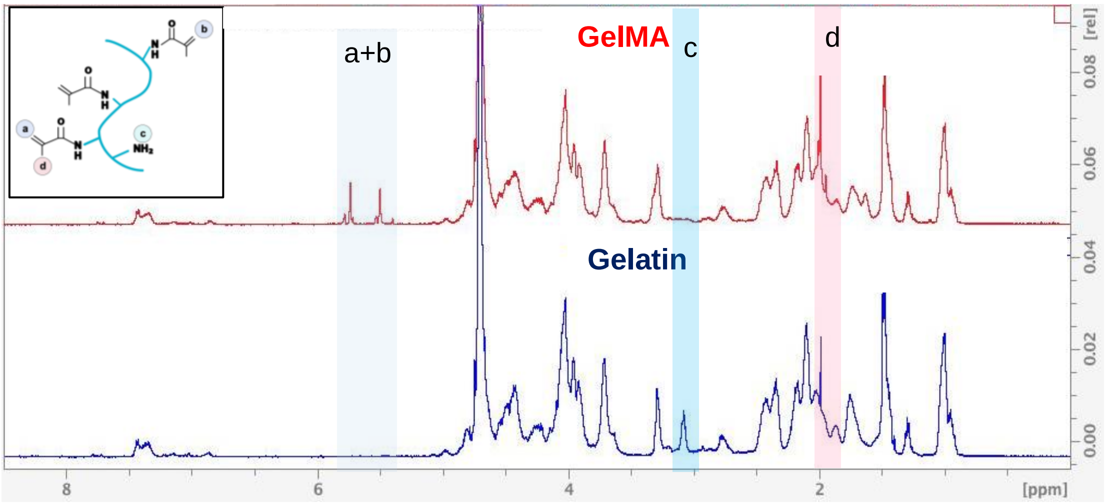

Figure S3: ^{1}H-NMR Spectra of GelMA and Gelatin. The upper-left panel depicts the corresponding chemical bonds.

4- Cell-laden GelMA fabrication

hMSC-laden GelMA hydrogels were fabricated following the procedures outlined in Section 4.7 of the main manuscript. Supplementary Figure 4 provides a schematic representation of the described process.

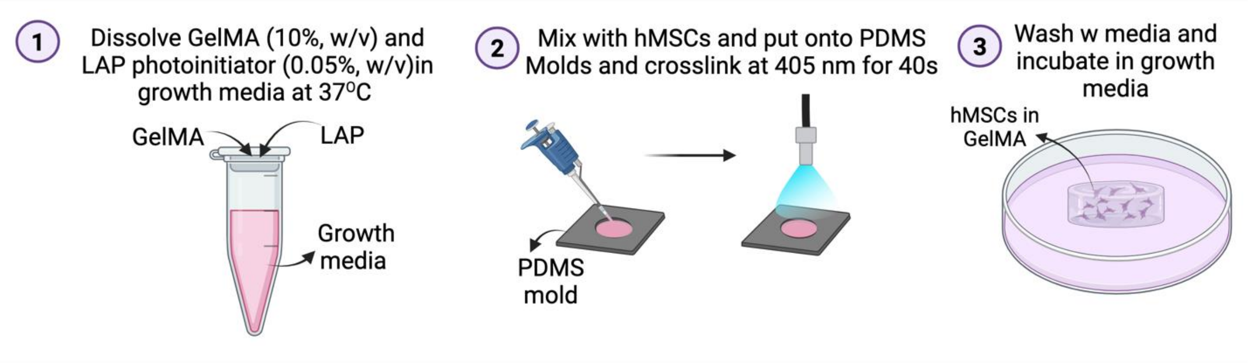

Figure S4: Schematic illustration detailing the fabrication process of GelMA hydrogels laden with hMSCs.

5- Surface area-to-volume ratio

The optical images in Figure 2A, used for calculating the average particle size of GelMPs, were employed to determine the total surface area and volume provided by GelMPs in each group. The calculations are summarized in Table S1, offering a clear depiction of the anticipated relationship between particle size and surface-to-volume ratio. GelMPs < 100, with an average particle size of 67 ± 20 µm, exhibit a higher surface area-to-volume ratio, providing more surface area for cell proliferation compared to larger-sized GelMPs.

Table S1. Comparison of the total surface area, total volume, and surface area-to-volume ratio of GelMPs samples.

Samples	Average Particle Size (µm)	Total Surface area ($\times10^6$ µm^2)	Total Volume ($\times10^6$ µm^3)	Surface Area/ Volume (1/µm)
GelMPs<100	67 ± 20	1.3	28.1	0.046
100<GelMPs<200	115 ± 16	1.7	59.8	0.029
GelMPs>200	204 ± 38	1.9	102.2	0.019
